# A randomised controlled trial on effectiveness and feasibility of sport climbing in Parkinson’s disease

**DOI:** 10.1038/s41531-021-00193-8

**Published:** 2021-06-10

**Authors:** Agnes Langer, Sebastian Hasenauer, Anna Flotz, Lucia Gassner, Rochus Pokan, Peter Dabnichki, Laurenz Wizany, Jakob Gruber, Dominik Roth, Sarah Zimmel, Marco Treven, Michaela Schmoeger, Ulrike Willinger, Walter Maetzler, Heidemarie Zach

**Affiliations:** 1 Department of Neurology, Medical University of Vienna, Vienna, Austria; 2 School of Engineering, RMIT University, Melbourne, VIC Australia; 3 Department of Sport Physiology, Institute of Sports Sciences, University of Vienna, Vienna, Austria; 4 Department of Emergency Medicine, Medical University of Vienna, Vienna, Austria; 5 Department of Neurology, Christian-Albrechts-University of Kiel, Kiel, Germany

**Keywords:** Parkinson's disease, Basal ganglia, Parkinson's disease, Rehabilitation, Outcomes research

## Abstract

Physical activity is of prime importance in non-pharmacological Parkinson’s disease (PD) treatment. The current study examines the effectiveness and feasibility of sport climbing in PD patients in a single-centre, randomised controlled, semi-blind trial. A total of 48 PD patients without experience in climbing (average age 64 ± 8 years, Hoehn & Yahr stage 2–3) were assigned either to participate in a 12-week sport climbing course (SC) or to attend an unsupervised physical training group (UT). The primary outcome was the improvement of symptoms on the Movement Disorder Society-Sponsored Revision of the Unified Parkinson’s Disease Rating Scale part III (MDS-UPDRS-III). Sport climbing was associated with a significant reduction of the MDS-UPDRS-III (−12.9 points; 95% CI −15.9 to −9.8), while no significant improvement was to be found in the UT (−3.0 points; 95% CI −6.0 to 0.1). Bradykinesia, rigidity and tremor subscales significantly improved in SC, but not in the unsupervised control group. In terms of feasibility, the study showed a 99% adherence of participants to climbing sessions and a drop-out rate of only 8%. No adverse events occurred. This trial provides class III evidence that sport climbing is highly effective and feasible in mildly to moderately affected PD patients.

## Introduction

Parkinson’s disease (PD) is a chronic progressive neurodegenerative disease, characterised by cardinal motor signs such as bradykinesia, rigidity and tremor. The chronic and progressive course of the disease requires a multimodal therapeutic approach. All types of treatments currently available provide only symptomatic relief, aiming at maintaining the highest possible level of functionality^1,2^. Besides pharmacological and invasive therapies, exercise is one of the fundamental pillars in PD treatment. It is widely known today that physical exercise has the ability to improve motor symptoms in PD^2–5^. Numerous studies on treadmill training, Nordic walking, cycling and resistance training have already revealed significant positive effects in this respect^1,6–13^. Nearly all types of exercise yield general therapeutic benefits, nevertheless, certain types of exercise are particularly effective on PD symptoms in the targeted body parts^14–16^. Although there is some evidence of carry-over effects to other parts of the body when one body part is trained^8^, it is generally accepted that the area being trained benefits the most. It seems, therefore, reasonable to pursue a whole-body training approach in PD treatment.

In addition to the undisputable symptomatic relief provided by physical activity, there is emerging evidence of potential disease-modifying effects demonstrated both at the cellular level through improvement of neuroplasticity and at the behavioural level resulting in physiological, functional and clinical improvement^1,17–20^. Randomised trials have shown promising results in the light of disease-modifying effects of exercise on the course of PD^21,22^. Further results from randomised controlled trials are currently in progress and eagerly awaited^23,24^.

Any success of exercise is determined by a regular routine, which requires a high level of motivation from the patient^25^. Engaging and motivating sports are essential to overcome apathy; a state of mind that prevails in patients suffering from PD and interferes with long-term adherence to physical exercise^26–30^. To widen the range of attractive sports for PD patients, alternative sports such as dancing, boxing, Tai Chi and yoga are currently on the rise and have already shown substantial effects on motor symptoms^31–36^.

**Fig. 1:**
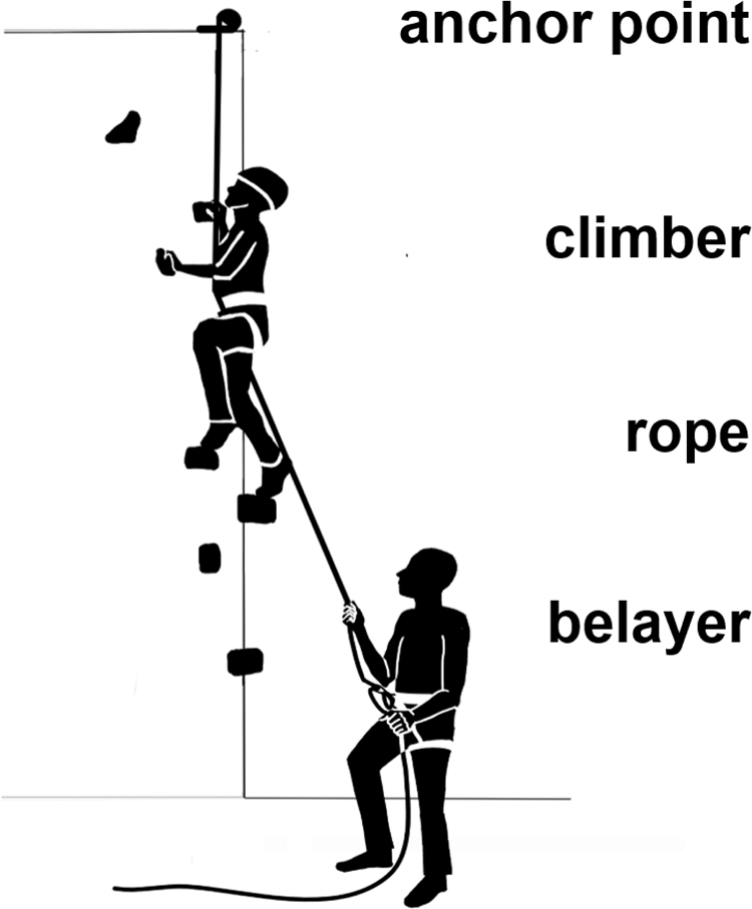
Schematic depiction of the top-rope climbing setup. The climber is secured by the belayer via the rope, which is fixed to an anchor point at the top of the wall. The rope minimises the climber’s fall distance in the event of a fall.

Climbing has great potential to soon make it onto the list of recognised attractive and effective sports for PD patients. It is performed in different ways: as boulder climbing, lead climbing, or top-rope climbing (see Fig. 1). In general, climbing (and especially top-rope climbing) is considered a safe sport and a type of exercise with a comparatively low risk of injury^37^, as shown in diverse (non-PD) cohorts^38–46^. Climbing is known to generally improve physical fitness, strength, posture, balance and flexibility^38,44,47,48^. It often requires reaching for distant holds and subsequently forces climbers to extend the range of motion. This goes in line with the PD-specific therapeutic “BIG” concept of emphasizing large movements and seems, therefore, to be particularly valuable for PD patients^49,50^. Climbing in neurorehabilitation programmes is already established as a therapeutic option for patients suffering from multiple sclerosis, cerebellar ataxia, traumatic brain injury and stroke^40,41,43,51–53^. Despite the lack of clinical trials, climbing (especially boulder climbing) is already implemented in various PD rehabilitation programmes^54,55^.

This is a randomised controlled, semi-blind trial evaluating the effects of climbing in PD patients. Top-rope climbing was investigated in terms of effectiveness and feasibility in a 12-week intervention with mild to moderately affected PD patients without prior climbing experience.

## Results

We screened 93 PD patients who expressed general interest to participate in the trial, to eventually include 48 climbing-naive patients. More detailed characteristics of all study participants are shown in Table 1. No significant differences at baseline were identified with regards to age, gender and Hoehn & Yahr (H&Y) stage between the sport climbing group (SC) and the active control group (unsupervised physical training group, UT). Two participants of the SC did not finish the trial (one due to a newly diagnosed prostatic cancer, the other one due to a lack of motivation).

**Table 1:** Demographics and clinical characteristics.

	SC	(*n* = 24)	UT	(*n* = 24)
Age (yr), mean (range)	65	(45–78)	64	(49–78)
Sex, *n* (%)				
Female	10	(42)	8	(33)
Male	14	(58)	16	(67)
Disease duration, months since diagnosis (range)	77	(2–144)	63	(2–180)
Hoehn & Yahr stage, *n* (%)				
2	20	(83)	22	(92)
3	4	(17)	2	(8)
MDS-UPDRS-III score, mean (SEM)	37.9	(2.2)	34.2	(2.9)
Patients on dopaminergic therapy, *n* (%)	24	(100)	23	(96)
LEDD, mg (range)	554	(200–1365)	609	(0–1464)
Patients with deep brain stimulation, *n* (%)	1	(4)	1	(4)
MMSE score, mean (SEM)	29.3	(0.2)	29.2	(0.2)

*SC* sport climbing group, *UT* unsupervised physical training group, *MDS-UPDRS-III* motor part of the Movement Disorder Society-Sponsored Revision of the Unified Parkinson’s Disease Rating Scale part III (score 0–132; higher scores indicate worse functioning), *SEM* standard error of the mean, *LEDD* levodopa equivalent daily dose per day, *MMSE* Mini-Mental State Examination (score 0–30; lower scores indicate worse functioning). Hoehn & Yahr stage (score 0–5). Data are mean (range, percentage), unless indicated otherwise.

### Physical activity in the control group

The 24 participants of the UT were asked to report their regular exercise routines in weekly telephone calls. Twenty participants returned their training logs at the end of the trial. A mean of 117 min of vigorous activities, such as jogging, cycling and skiing, was reported. A mean of 272 min per week of moderate activities, such as physical therapy, yoga and swimming was reported, resulting in a total of 389 min of physical activity. This amounts to approximately twice as much time on weekly physical activity as recommended by the first World Health Organisation (WHO) or the European Physiotherapy Guidelines for PD patients^56,57^. In addition, the patients reported an average of 75 min per week spent on low-impact activities (leisurely walking, easy housework/gardening, balance exercises).

### Clinical outcomes

**Fig. 2:**
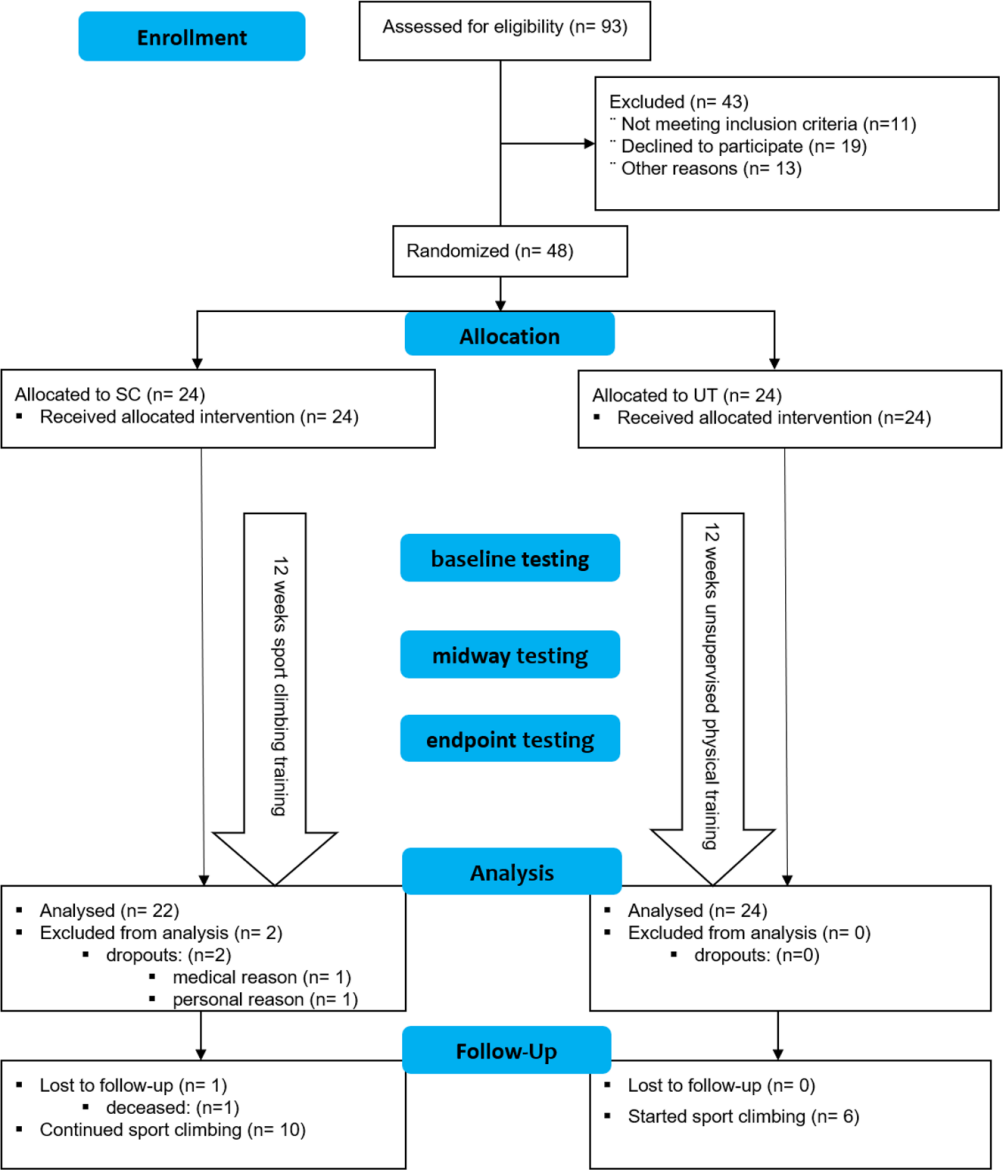
Trial flowchart. In all, 93 patients were screened to meet the predefined necessary number of participants (24 participants in each group for a total of 48 participants). Other reasons for exclusion before randomisation: organisational reasons (timing issues, distance to climbing facility), unwilling to be randomised (preference for either intervention or control group). SC, sport climbing group; UT, unsupervised physical training group.

At baseline the mean Movement Disorder Society-Sponsored Revision of the Unified Parkinson’s Disease Rating Scale part III (MDS-UPDRS-III) did not significantly differ between the two groups (SC: 37.5 points, 95% CI [32.5, 42.5], UT: 34.0 points; 95% CI [27.8, 40.2], mean difference 3.5 points, 95% CI [−4.3, 11.3]). Within a period of 6 weeks, there was a significant improvement of motor symptoms in the SC, reflected by a decrease of the MDS-UPDRS-III by 9.2 points (95% CI [−11.7, −6.8]), but not in the UT (−1.7 points; 95% CI [−5.4, 2.0]; Fig. 2 and Table 2). Within a period of 12 weeks, there was a significant improvement of motor symptoms in the SC, reflected by a decrease of the MDS-UPDRS-III by 12.9 points (95% CI [−15.9, −9.8]), but not in the UT (−3.0 points; 95% CI [−6.0, 0.1]; Fig. 2 and Table 2). Being part of the SC significantly predicted MDS-UPDRS-III scores (coeff. −9.9; *p* < 0.0001, *R*^2^ = 0.34) as compared to the UT, according to the regression model.

**Table 2:** Clinical outcomes.

	BASE		MID (6 weeks)		END (12 weeks)		Absolute change (within-group) from BASE to MID		Absolute change (within-group) from BASE to END		SC vs. UT
MDS-UPDRS-III	Mean	95% CI	Mean	95% CI	Mean	95% CI	Mean	95% CI	Mean	95% CI	*p*
SC (*n* = 22)	37.5	32.5, 42.5	28.3	22.8, 33.7	24.6	20.7, 28.5	−9.2	−11.7, −6.8	−12.9	−15.9, −9.8	<0.001
UT (*n* = 24)	34.0	28.2, 41.0	32.9	26.3, 39.5	31.0	26.6, 36.8	−1.7	−5.4, 2.0	−3.0	−6.0, 0.1	
MDS-UPDRS-III_brad_											
SC	18.3	15.7, 20.9	13.9	10.9, 16.9	13.1	10.6, 15.7	−4.5	−6.4, −2.5	−5.2	−6.8, −3.6	0.003
UT	17.8	15.6, 20.7	17.1	14.2, 20.0	15.9	13.9, 18.8	−1.0	−2.7, 0.6	−1.8	−4.0, 0.4	
MDS-UPDRS-III_rig_											
SC	6.0	4.6, 7.4	4.7	3.3, 6.2	4.2	2.9, 5.6	−1.3	−2.3, −0.3	−1.8	−2.6, −1.0	0.016
UT	5.3	4.0, 6.7	5.3	3.9, 6.8	5.5	4.3, 7.0	0.0	−1.1, 1.1	−0.3	−0.6, 1.1	
MDS-UPDRS-III_trem_											
SC	9.5	6.7, 12.3	6.9	4.5, 9.4	4.6	2.6, 6.6	−2.6	−4.0, −1.2	−4.9	−7.0, −2.8	0.001
UT	7.6	5.0, 10.5	7.2	4.8, 9.6	6.6	4.6, 8.5	−0.6	−2.4, 1.3	−1.0	−2.5, 0.4	

*BASE* Baseline (before intervention), *MID* visit after 6 weeks of intervention, *END* visit after 12 weeks of intervention (end of trial); *SC* sport climbing group, *UT* unsupervised physical training group, *MDS-UPDRS-III* Movement Disorder Society-Sponsored Revision of the Unified Parkinson’s Disease Rating Scale part III, scale for the assessment of Parkinson’s symptoms (score ranges from 0 to 132), *MDS-UPDRS-III*_*brad*_ severity of bradykinesia (14 items; items 4–11 and 14; score ranges from 0 to 56), *MDS-UPDRS-III*_*rig*_ severity of rigidity (5 items; item 3; score ranges from 0 to 20), *MDS-UPDRS-III*_*trem*_ severity of tremor (10 items; items 15–18; score ranges from 0 to 40). Data are mean and 95% of confidence interval (95% CI).

Climbing significantly improved bradykinesia (MDS-UPDRS-III_brad_) by a mean of 4.5 points (95% CI [−6.4, −2.5]) within 6 weeks, and by a mean of 5.2 points (95% CI [−6.8, −3.6], relative improvement 28%) within 12 weeks. Unsupervised physical training did not significantly improve the MDS-UPDRS-III_brad_ (mean change after 6 weeks −1.0 points, 95% CI [−2.7, 0.6], mean change after 12 weeks −1.8 points, 95% CI [−4.0, 0.4]). Climbing significantly predicted MDS-UPDRS-III_brad_ scores (coeff. −3.3; *p* = 0.016, *R*^2^ = 0.13).

Within 6 weeks, climbing significantly improved rigidity (MDS-UPDRS-III_rig_) by a mean of 1.3 points (95% CI [−2.3, −0.3]. Within 12 weeks, climbing significantly improved the MDS-UPDRS-III_rig_ by a mean of 1.8 points (95% CI [−2.6, −1.0], relative improvement 30%). Independent physical training did not significantly improve the MDS-UPDRS-III_rig_ (mean difference after 6 weeks 0.0 points, 95% CI [−1.1, 1.1], mean difference after 12 weeks 0.3 points, 95% CI [−0.6, 1.1]). Again, climbing significantly predicted MDS-UPDRS-III_rig_ scores (coeff. −2.0; *p* = 0.001, *R*^2^ = 0.22).

Climbing significantly improved tremor (MDS-UPDRS-III_trem_) by a mean of 2.6 points (95% CI [−4.0, −1.2] within 6 weeks, and by a mean of 4.9 points (95% CI [−7.0, −2.8], relative improvement 51%) within 12 weeks. Independent physical training did not significantly improve MDS-UPDRS-III_trem_ (mean difference after 6 weeks −0.6 points, 95% CI [−2.4, 1.3], mean difference after 12 weeks −1.0 points, 95% CI [−2.5, 0.4]). Climbing significantly predicted MDS-UPDRS-III_trem_ scores (coeff. −3.8; *p* = 0.003, *R*^2^ = 0.18).

### Dopaminergic medication

During the 12-week study period, levodopa equivalent daily dose (LEDD; mg) was increased in two participants of the SC. Specifically, in one male patient (64 yr, disease duration: 144 months, MDS-UPDRS III: 35 points) LEDD was increased by 105 mg and in one female patient (72 yr, disease duration: 72 months, MDS-UPDRS III: 42 points) by 100 mg. In the UT the LEDD of a male patient (65 yr, disease duration 65 months, MDS-UPDRSIII: 13 points) was reduced by 50 mg. No relevant change occurred in the main results after excluding these three participants from the analysis (data not shown).

### Feasibility

In all, 92% of the SC (22 out of 24 participants) and 100% of the UT (24 out of 24) completed the trial. In the SC, adherence to climbing was excellent with 99% course participation (only 3 out of 264 climbing sessions were missed out), which amounts to an excellent adherence of 99%. No adverse events occurred in either the SC or the UT.

A follow-up telephone interview with all 22 SC participants who completed the intervention was performed 12 ± 0.5 months after the end of the study. One participant had died of heart failure (unrelated to climbing), resulting in a total of 21 participants who were interviewed 12.5 months after the end of the intervention. Of these 21 participants, 10 (48%) continued with climbing in newly established public PD climbing courses. Reasons given for why participants continued climbing after the end of the study were the following (in descending order): a feeling of improved mobility and posture (n = 4, 40%), pure enjoyment (n = 3, 30%), better overall fitness (n = 2, 20%), and well-being (n = 1, 10%). Participants who stopped climbing after the trial gave various reasons for their behaviour, such as the arrival of new health problems unrelated to climbing (*n* = 4, 40%), time constraints (*n* = 2, 20%), the cost of climbing courses (*n* = 2, 20%), the distance between home and a climbing facility (*n* = 1, 10%), a feeling that climbing has no effects on PD symptoms (*n* = 1, 10%) and a complete loss of interest in climbing (*n* = 1, 10%). Four participants (36%) expressed a desire, based on positive experiences made during the intervention, to continue with climbing beyond 12 weeks, but the hindrances mentioned above made them do otherwise. In the UT, 6 (25%) participants started climbing after completing the trial.

## Discussion

In this randomised controlled, semi-blind trial we investigated the effectiveness and feasibility of a 12-week sport climbing course in comparison to unsupervised physical training on motor symptoms in PD patients without prior climbing experience. This trial revealed two main findings. Firstly, motor symptoms improved significantly and substantially due to climbing. Secondly, climbing proved to be a feasible exercise for PD patients without prior climbing experience.

Climbing significantly improved PD motor symptoms by a mean of 12.9 points on the MDS-UPDRS-III scale in the medical ON-state. The MDS-UPDRS-III scores of the SC were significantly better (a mean of 6.4 points) than those of the participants of the UT. The improvement after attending the 12-week climbing course most likely also translates to a highly relevant improvement of motor symptoms in daily life of PD patients, since an improvement of 3.5 points on the MDS-UPDRS-III scale is considered a clinically relevant change^58^. This immense effect can be explained by the fact that climbing training is a special combination of resistance training, balance, flexibility and coordination training. Resistance training has been proven to be particularly effective in improving bradykinesia and rigidity^6,7,10,36^. Balance, flexibility and coordination training are highly effective in improving functional mobility, postural control and dual-task ability—all of which are known to significantly reduce the risk of falls^34,38,47,59–62^.

A comparable improvement in the MDS-UPDRS-III, as shown in the study at hand, is found in only a few studies with comparable design and training intensity. In an elegant trial on tango dancing lasting over 12 months, the MDS-UPDRS-III improved by 13 points^63^. As is climbing, so is tango an equally demanding sport that requires complex movements, balance, flexibility, endurance and coordination. Together with the results presented here, this indicates that whole-body workouts are extremely effective in improving motor symptoms in PD, potentially even beyond those parts of the body directly trained.

To further support the importance of a complex whole-body workout to achieve best results for patients suffering from PD, the following observation must be brought to the fore: endurance sports, which predominantly target leg movements, seem to have smaller (albeit significant and clinically meaningful) effects on MDS-UPDRS-III than resistance training, as shown in excellent studies on high-intensity treadmill exercise and cycling^1,12,64^. Of all the endurance sports mentioned in this context, Nordic walking is reported to achieve the best amelioration of motor symptoms. The additional pronounced arm movements that come along with the sport may give reason for the outstanding effectivity rates related to Nordic walking^13^. However, compared to whole-body workouts such as conventional physiotherapy, Tai Chi, Lee Silverman Voice Treatment-BIG (LSVT-BIG), stretching and resistance training, all of which are to be perceived as highly valuable therapeutic strategies, the current trial even revealed superior effects on motor symptoms as a result of climbing. A possible explanation could be the above-mentioned combination of different training components that are unique to climbing^34,49,64–66^.

Sport climbing also substantially reduced cardinal motor features of PD and thus suggests a symptomatic effect.

Climbing substantially improved *bradykinesia*, with a mean improvement of 28% after 12 weeks of climbing. Interestingly, a mean improvement of 24% was already even measurable after 6 weeks (only half-way through the course; see Fig. 2c). This observation suggests that even a short period of time spent on climbing can reduce bradykinesia. Knowledge of this kind is relevant as PD patients with reduced bradykinesia can perform faster compensatory movements and thus benefit from fewer falls^3,9^. Reduced bradykinesia is known to have positive effects on sleep quality, allows PD patients to be more independent in daily life, and generally improve quality of life^67–73^. The resistance training element to be found in sport climbing is probably most responsible for the improvement in bradykinesia^44,47^. This observation is consistent with prior studies in the field that have shown bradykinesia improvement (be it in leg or arm) after resistance training of the respective extremities^7,8,36,74,75^.

Sport climbing also proved to be highly effective in terms of *rigidity* and showed a mean improvement of 1.3 points after 6 weeks (21%) and 1.8 points (30%) after 12 weeks. Participants explicitly reported feeingl less rigid after climbing. A possible driver of this effect may be found in the resistance training components of all four limbs that comes naturally along with sport climbing. Previous studies have already reported improvement of rigidity after resistance exercises^8,10,76^. In comparison to LSVT-BIG and Tai Chi, exercises that both focus on smooth maximum-amplitude movements and less prominently feature resistance training components^34,49^, climbing seems to have better effects on rigidity. All in all, PD patients seem to particularly benefit from sport climbing in terms of rigidity by the somewhat unique combination of full-body resistance training, coordination training and high-amplitude movements that constitute climbing.

In the light of percentages, *tremor* responded best to the intervention (51% improvement), followed by rigidity (30% improvement) and bradykinesia (28% improvement). This is particularly remarkable as fatigue-induced tremor occurs after exercise, even in healthy subjects^77,78^. For this study, however, participants were not immediately evaluated in terms of tremor after the various climbing sessions. A possible explanation for this long-term positive effect on tremor could be found in a general reduction of the participants’ stress level during and after climbing^79^. A similar effect on tremor has been shown in studies on mindfulness and yoga training, in which the authors attribute the effect to improved body awareness and sensory feedback through physical exercise and the components of resistance training^14–16,61,80^.

In the UT, the level of unsupervised physical training was high and almost two times higher as recommended by the WHO and the European Physiotherapy Guidelines for PD patients. Participants in the UT were able to maintain stable scores on the MDS-UPDRS-III and the subscales bradykinesia, rigidity and tremor in the current study. The stability of scores is most likely derived from the high level of physical activity during the intervention. In the study at hand, general physical training in the UT led to a general halt of further disease progression. Within 12 weeks, a worsening of approximately 1 point on the MDS-UPDRS-III scale is to be expected in the natural course of the disease^13,81^. In our view, the large difference in the main outcomes between SC and UT, despite the high level of activity in our control group, must be understood as clear evidence for the remarkable effectiveness climbing has on PD patients.

Although climbing is commonly mistaken as an extreme sport, we could show in our trial that it is in fact a very safe full-body workout. Despite its image of being a risky and strenuous kind of sport, sport climbing proved to be a feasible training method for PD patients. In consideration of the high prevalence of osteoporosis in our elderly study population, our participants performed top-rope climbing, known as the safest and most elaborate form of climbing. We observed excellent adherence in the course of the intervention, comparable to other exercise-based interventions of the kind, such as treadmill training, resistance exercises and cycling^1,5,60^.

Our trial shows that climbing is a fun sport, rich in physical and mental challenges and with the potential to be highly motivational for PD patients to engage in physical activities in the long run. The high adherence, the low drop-out rate, of 2 participants, and the positive feedback to the intervention prove this observation right. The participant who dropped out due to newly diagnosed prostatic cancer expressed great interest in resuming the trial after cancer treatment. Since the cancer treatment exceeded the study period in time, the respective participant could not finish the trial. A high percentage of participants in the control group began climbing after the end of the trial. Climbing is both attractive and feasible for PD patients. Participants named several factors as to why they were not willing to continue climbing after the intervention, such as lack of time, financial aspects and the lack of nearby climbing gyms. The availability of climbing gyms is less of a problem in cities as opposed to rural areas. Since PD climbing proved to be an effective method of therapy, one could consider that climbing costs could even be covered by health insurances in the future, thus removing potential financial barriers for PD patients.

This study faces some limitations. Firstly, our main outcome measure was the MDS-UPDRS-III scale score, which is influenced by pharmacological treatment. Therefore, we discouraged medication changes during the course of the study to minimise this potential confounder. For a total of 2 participants in the SC and 1 participant in the UT, minor optimisation of LEDD during the study was unavoidable. The results remained the same in the pooled calculated outcome measures with and without the 3 participants. We are, therefore, confident that treatment adaptations did not significantly influence our results. Secondly, while there might be an inclusion bias due to the fact that only participants with a positive attitude towards exercise and climbing expressed interest in this study, we only included participants without previous climbing experience. Our results suggest that starting to climb is feasible for PD patients at any age and without any prior experience in this sport area. Thirdly, our follow-up assessment (i.e. interview) did not include another clinical assessment. Additionally, although this trial included a considerable number of patients, future studies would add important insights by evaluating the long-term effect of sport climbing on motor symptoms and the translation to daily life in an even larger cohort of PD patients. Finally, the control group was not directly supervised. However, the aim was to imitate a real-life setting. In addition, regular telephone checks and training logs were used. Eventually, the participants performed almost twice as much exercise as recommended, which can well explain the lack of (expected) deterioration of PD symptoms in this group within the study period. Considering the fact that the UT demonstrated a surprisingly high level of physical activity, a John Henry effect—the phenomenon of unconscious or conscious effort on the part of the control group to compensate for the difference to the experimental group—possibly occurred^82^. Nevertheless, since the motor symptoms of the control group did not measurably improve, any confounding effect was too small to make a statistically significant difference.

In conclusion, this randomised controlled trial shows that sport climbing is a feasible sport even for inexperienced PD patients and that it significantly improves motor symptoms in PD. The effect was greater than that of most previous studies on other forms of exercise or classical physiotherapy. In contrast to climbing, unsupervised physical training in the control group only stabilised but did not improve, motor symptoms^2,5^. Moreover, climbing turned out to be safe, highly feasible as well as motivating to the patients. The results presented here provide class III evidence for the efficiency of climbing to reduce motor deficits in PD and demonstrate that climbing is also a highly attractive sport for PD patients.

## Methods

### Registration and informed consent

The study was approved by the ethical committee of the Medical University of Vienna (No. 1369/2017) and registered within the U.S. National Library of Medicine (No: NCT04569981). It was performed according to the standards of the 1964 Declaration of Helsinki. All participants gave written informed consent before their inclusion.

### Design

This is a single-centre, randomised controlled, semi-blind trial, comparing the effect of sport climbing with unsupervised physical training on motor symptoms in PD over a period of 12 weeks. All patients who provided written informed consent to participate in this trial were assigned a number before being randomly allocated to one of the two groups of equal sample size by using a table-generated permuted block randomisation method^83^. The randomisation ratio of intervention was 1:1 to either the SC (*n* = 24) or the UT (*n* = 24).

The SC followed a 12 week, 90 min per week supervised top-rope sport climbing course in an indoor climbing gym with an instructor-to-participant ratio of 1:3–4. The participants were trained in top-rope climbing: the most common style at indoor climbing walls, which involves a “belayer”, i.e. a person standing on the ground securing the rope holding the climber. The rope runs from the belayer through carabiners which are connected to an anchor system at the top of the route and back down to the climber (see Fig. 1). Usually, the instructor served as the belayer. However, if participants wanted to do so, they could also act as belayers (under the supervision of the instructor) while other participants took their turn to climb.

Participants of the UT received an individual session of basic education based on the “European Physiotherapy Guidelines for Parkinson’s Disease” and on the WHO recommendations for what makes an active lifestyle including 150 min per week of moderate or 75 min per week of vigorous aerobic physical activities, resistance training twice a week and balance exercises thrice a week^56,57^. The participants were instructed to follow the given recommendations independently and without supervision, and to complete and return a training log. To motivate and to ensure compliance with the study design, UT participants were given regular calls by study team members every 7–10 days.

UT participants were given the opportunity to join a free climbing lesson after completion of the trial and to receive regular updates on available PD climbing groups and courses in case of interest.

Participants of both groups were discouraged from changing medication and deep brain stimulation settings throughout the study period. However, to provide a realistic clinical situation, patients were allowed to change treatment under the supervision of their treating physicians, if necessary. LEDD was assessed at each visit^84^.

### Participants

**Fig. 3:**
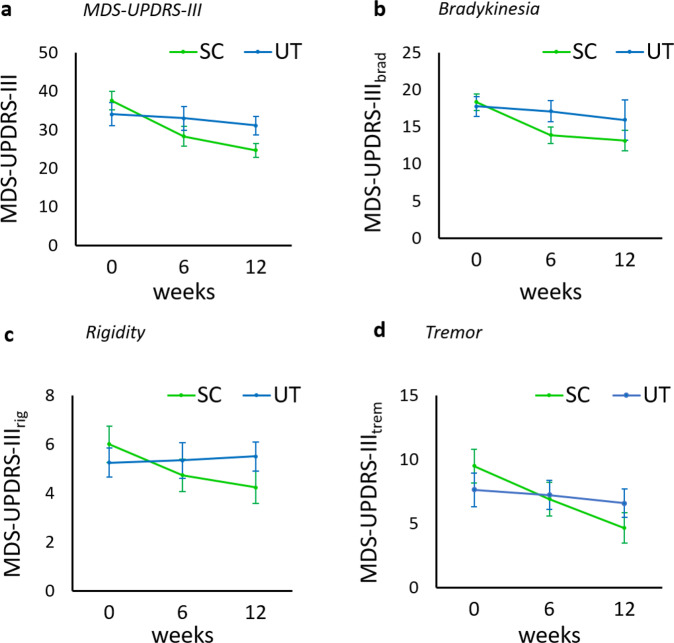
The effect of sport climbing on Parkinson’s disease motor symptoms. Data are mean and standard error of the mean (SEM). SC, sport climbing group (green lines); UT, unsupervised physical training group (blue lines); MDS-UPDRS-III, Movement Disorder Society-Sponsored Revision of the Unified Parkinson’s Disease Rating Scale part III (score ranges from 0 to 132); MDS-UPDRS-III_brad_, severity of bradykinesia (14 items on the MDS-UPDRS-III; items 4–11 and 14; score ranges from 0 to 56); MDS-UPDRS-III_rig_, severity of rigidity (5 items on the MDS-UPDRS-III; item 3; score ranges from 0 to 20); MDS-UPDRS-III_trem_, severity of tremor (10 items on the MDS-UPDRS-III; items 15–18; score ranges from 0 to 40). **a** The effect of climbing on the *total* MDS-UPDRS-III score at baseline (BASE), after 6 weeks (MID) and after 12 weeks (END) compared to unsupervised physical training. Climbing significantly reduced total score on the MDS-UPDRS-III after 12 weeks, while unsupervised physical training stabilised motor symptoms. The cardinal symptoms are displayed in (**b**) (bradykinesia), (**c**) (rigidity) and (**d**) (tremor). All cardinal symptoms significantly improved in the climbing group and stabilised in the UT.

From June 2018 to May 2019, we included 48 climbing-naive PD patients, diagnosed according to the UK Brain Bank criteria^85^ of mild or moderate disease severity (H&Y stage 2–3), and stable dopaminergic medication for at least 1 month (see Fig. 3). Exclusion criteria were a history of stroke, severe orthopaedic, visual or hearing problems as judged by the investigator and a Mini-Mental State Examination (MMSE) score <24^86^. Participants were made aware of the trial via their treating neurologists, and via diverse local media channels. All data were collected at the outpatient movement disorders clinic at the Department of Neurology, Medical University of Vienna.

### Measurements

We investigated the total scores of the MDS-UPDRS-III, determined by movement disorder specialists who were blinded to the participants’ allocation at baseline (BASE) after 6 weeks (MID) and after 12 weeks at the end of the intervention (END) in the participants’ best ON-state. Further outcomes included the following subscales of the MDS-UPDRS-III and were determined in the same manner as the primary outcome:

1. Bradykinesia (MDS-UPDRS-III_brad_: 14 scores on items 4–11 and 14; 0–56 points).
2. Rigidity (MDS-UPDRS-III_rig_: 5 scores on item 3; 0–20 points).
3. Tremor (MDS-UPDRS-III_trem_: 10 scores on items 15–18; 0–40 points).

Furthermore, within the SC we assessed feasibility outcomes including the willingness to continue climbing beyond the trial. Adherence outcomes such as *course participation*, i.e. the number of missed climbing sessions (%) and *drop-out rates* (%), as well as climbing-related *adverse events* (injuries requiring medical attention and/or immobilisation, e.g. fractures, strains or sprains) were documented throughout the trial.

Continuation of climbing after the trial was evaluated via follow-up telephone interviews 12 ± 0.5 months after the end of the study.

### Statistical analysis

Sample size considerations were based on a minimal clinically relevant effect of the intervention represented by an absolute difference in MDS-UPDRS-III of at least 4 points between the two groups^58^. We expected a standard deviation of the difference of 5 points for both groups, based on previous experience. Based on these calculations, 21 subjects needed to be included in each group (42 in total) to show a difference with a power of 0.8 based on a probability of error of the first kind of 0.05. To allow for loss-of-follow-up, as well as potential effects of the study design, we decided to include 24 subjects each.

For the analysis of the primary outcome (MDS-UPDRS-III), we tabulated results by baseline vs. after 12 weeks as well as by group (SC vs. UT). We then calculated for each group separately absolute mean differences between baseline (BASE) and after 12 weeks (END) with robust 95% confidence intervals. We formally tested for an influence of group assignment on MDS-UPDRS-III using a linear regression model. Mean score of MDS-UPDRS-III after 12 weeks served as the dependent variable and the assignment to the intervention group as an indicator-covariate. We report both coefficients and *p*-values derived from the *t*-statistic of the covariate. Furthermore, we calculated the relative change between baseline and after 12 weeks. The analyses of the subscales MDS-UPDRS-III_brad_, MDS-UPDRS-III_rig_ and MDS-UPDRS-III_trem_ followed the primary outcome analysis. A two-sided *p*-value below 0.05 was considered statistically significant. STATA 16 (Stata Corp, College Station, TX) was used^87^.

### Reporting summary

Further information on research design is available in the Nature Research Reporting Summary linked to this article.

## Supplementary information

Reporting summary.

## Data Availability

Data are available on request to the corresponding author.

## Code availability

No previously unreported custom computer code or algorithm was used to generate results in this study.
