# Supplementary material for: A randomised controlled trial on effectiveness and feasibility of sport climbing in Parkinson’s disease
Source: NPJ Parkinsons Dis. 2021 Jun 10;7:49. doi: 10.1038/s41531-021-00193-8 (PMC8192917; doi:10.1038/s41531-021-00193-8)
Supplement: Supplementary file 1 — Reporting summary. [file 41531_2021_193_MOESM1_ESM.pdf]

nature research

Corresponding author(s): Dr. med. Univ. Heidemarie Zach, BSc
Medical University of Vienna, Dept. of Neurology
Waehringer Guertel 18-21
1090 Vienna, Austria
E-mail: heidemarie.zach@meduniwien.ac.at

Last updated by author(s): May 5, 2021

Reporting Summary

Nature Research wishes to improve the reproducibility of the work that we publish. This form provides structure for consistency and transparency in reporting. For further information on Nature Research policies, see our Editorial Policies and the Editorial Policy Checklist.

Statistics

For all statistical analyses, confirm that the following items are present in the figure legend, table legend, main text, or Methods section.

n/a	Confirmed	
☐	☒	The exact sample size (n) for each experimental group/condition, given as a discrete number and unit of measurement
☐	☒	A statement on whether measurements were taken from distinct samples or whether the same sample was measured repeatedly
☐	☒	The statistical test(s) used AND whether they are one- or two-sided<br>*Only common tests should be described solely by name; describe more complex techniques in the Methods section.*
☐	☒	A description of all covariates tested
☐	☒	A description of any assumptions or corrections, such as tests of normality and adjustment for multiple comparisons
☐	☒	A full description of the statistical parameters including central tendency (e.g. means) or other basic estimates (e.g. regression coefficient) AND variation (e.g. standard deviation) or associated estimates of uncertainty (e.g. confidence intervals)
☐	☒	For null hypothesis testing, the test statistic (e.g. F, t, r) with confidence intervals, effect sizes, degrees of freedom and P value noted<br>*Give P values as exact values whenever suitable.*
☒	☐	For Bayesian analysis, information on the choice of priors and Markov chain Monte Carlo settings
☒	☐	For hierarchical and complex designs, identification of the appropriate level for tests and full reporting of outcomes
☒	☐	Estimates of effect sizes (e.g. Cohen's d, Pearson's r), indicating how they were calculated

Our web collection on statistics for biologists contains articles on many of the points above.

Software and code

Policy information about availability of computer code

Data collection	No software was used for data collection.
Data analysis	STATA 16 (Stata Corp, College Station, TX) was used.

For manuscripts utilizing custom algorithms or software that are central to the research but not yet described in published literature, software must be made available to editors and reviewers. We strongly encourage code deposition in a community repository (e.g. GitHub). See the Nature Research guidelines for submitting code & software for further information.

Data

Policy information about availability of data

All manuscripts must include a data availability statement. This statement should provide the following information, where applicable:

- Accession codes, unique identifiers, or web links for publicly available datasets
- A list of figures that have associated raw data
- A description of any restrictions on data availability

Data are available on request to the corresponding author.

Field-specific reporting

Please select the one below that is the best fit for your research. If you are not sure, read the appropriate sections before making your selection.

☒ Life sciences ☐ Behavioural & social sciences ☐ Ecological, evolutionary & environmental sciences

For a reference copy of the document with all sections, see nature.com/documents/nr-reporting-summary-flat.pdf

Life sciences study design

All studies must disclose on these points even when the disclosure is negative.

Sample size	Sample size considerations were based on a minimal clinically relevant effect of the intervention represented by an absolute difference in MDS-UPDRS-III of at least 4 points between the two groups. We expected a standard deviation of the difference of 5 points for both groups, based on previous experience. Based on these calculations, 21 subjects needed to be included in each group (42 in total) to show a difference with a power of 0.8 based on a probability of error of first kind of 0.05. To allow for loss-of-follow up, as well as potential effects of the study design, we decided to include 24 subjects each.
Data exclusions	No data were excluded from the analysis.
Replication	not applicable
Randomization	All patients who provided written informed consent to participate in this trial were assigned a number before being randomly allocated to one of the two groups of equal sample size by using a table-generated permuted block randomisation method (85). Randomisation ratio of intervention was 1:1 to either the SC (n=24) or the UT (n=24).
Blinding	We investigated the total scores of the MDS-UPDRS-III, determined by movement disorder specialists who were blinded to the participants' allocation.

Reporting for specific materials, systems and methods

We require information from authors about some types of materials, experimental systems and methods used in many studies. Here, indicate whether each material, system or method listed is relevant to your study. If you are not sure if a list item applies to your research, read the appropriate section before selecting a response.

Materials & experimental systems

n/a	Involved in the study	
☒	☐	Antibodies
☒	☐	Eukaryotic cell lines
☒	☐	Palaeontology and archaeology
☒	☐	Animals and other organisms
☐	☒	Human research participants
☐	☒	Clinical data
☒	☐	Dual use research of concern

Methods

n/a	Involved in the study	
☒	☐	ChIP-seq
☒	☐	Flow cytometry
☒	☐	MRI-based neuroimaging

Human research participants

Policy information about studies involving human research participants

Population characteristics	We included 48 climbing-naïve PD patients, diagnosed according to the UK Brain bank criteria (87) of mild or moderate disease severity (Hoehn & Yahr (H&Y) stage 2-3), and stable dopaminergic medication for at least one month (please see figure 2). Exclusion criteria were a history of stroke, severe orthopaedic, visual or hearing problems as judged by the investigator and a Mini-Mental State Examination (MMSE) score <24 (88). P
Recruitment	Participants were made aware of the trial via their treating neurologists, and via diverse local media channels.
Ethics oversight	The study was approved by the ethical committee of the Medical University of Vienna (No. 1369/2017).

Note that full information on the approval of the study protocol must also be provided in the manuscript.

Clinical data

Policy information about clinical studies

All manuscripts should comply with the ICMJE guidelines for publication of clinical research and a completed CONSORT checklist must be included with all submissions.

Clinical trial registration	The study was registered within the U.S. National Library of Medicine (No: NCT04569981).
Study protocol	Study protocol is available on request to the corresponding author.
Data collection	This is a single-centre, randomised controlled, semi-blind trial, comparing the effect of sport climbing with unsupervised physical training on motor symptoms in PD over a period of 12 weeks. We investigated the total scores of the MDS-UPDRS-III, determined by movement disorder specialists who were blinded to the participants' allocation at baseline (BASE) after 6 weeks (MID) and after 12 weeks at the end of the intervention (END) in the participants' best ON-state. Continuation of climbing after the trial was evaluated via follow-up telephone interviews 12±0.5 months after the end of the study.
Outcomes	We investigated the total scores of the MDS-UPDRS-III, determined by movement disorder specialists who were blinded to the participants' allocation at baseline (BASE) after 6 weeks (MID) and after 12 weeks at the end of the intervention (END) in the participants' best ON-state. Further outcomes included the following subscales of the MDS-UPDRS-III and were determined in the same manner as the primary outcome:<br>1. bradykinesia (MDS-UPDRS-IIIbrad:14 scores on items 4-11 and 14; 0-56 points)<br>2. rigidity (MDS-UPDRS-IIIrig: 5 scores on item 3; 0-20 points)<br>3. tremor (MDS-UPDRS-IIItrem: 10 scores on items 15 to 18; 0-40 points)<br>Furthermore, within the SC we assessed feasibility outcomes including the willingness to continue climbing beyond the trial. Adherence outcomes such as course participation, i.e., number of missed climbing sessions [%] and drop-out rates [%] as well as climbing related adverse events (injuries requiring medical attention and/or immobilization, e.g., fractures, strains or sprains) were documented throughout the trial.<br>Continuation of climbing after the trial was evaluated via follow-up telephone interviews 12±0.5 months after the end of the study.
